# Dynamics of serum anion gaps with in-hospital mortality: Analysis of the multi-open databases

**DOI:** 10.1371/journal.pone.0302206

**Published:** 2024-04-16

**Authors:** Dong Eun Yang, Sua Jo, Dong Hyun Lee, Won Suk An, Min Jae Jeong, Minkook Son

**Affiliations:** 1 Department of Internal Medicine, Dong-A University College of Medicine, Busan, Republic of Korea; 2 Department of Hospital Medicine, Inha University Hospital, Incheon, Republic of Korea; 3 Department of Pulmonology and Intensive Care Medicine, Dong-A University College of Medicine, Busan, Republic of Korea; 4 Department of Physiology, Dong-A University College of Medicine, Busan, Republic of Korea; 5 Department of Data Sciences Convergence, Dong-A University Interdisciplinary Program, Busan, Republic of Korea; Sant Anna Hospital: Clinica Sant’Anna, SWITZERLAND

## Abstract

**Background:**

Few studies have investigated the relationship between the anion gap, including the corrected anion gap, and patient mortality in intensive care units (ICUs) without restricting the analysis to specific diseases or medical specialties. Our primary objective was to investigate the association between the anion gap and ICU mortality using multiple open-access databases.

**Methods:**

We identified 4229 subjects from the Medical Information Mart for Intensive Care IV (MIMIC-IV) database, whose entries were from between 2008 and 2019. For each patient, the anion gap and corrected anion gap were calculated, and the study sample was divided into tertile groups (T) according to these levels. The association between the anion gap and in-hospital mortality was assessed using hazard ratios (HRs) and 95% confidence intervals (CIs) derived from a multivariable-adjusted Cox proportional hazards model. Besides MIMIC-IV, we also incorporated study samples from two other databases (MIMIC-III and electronic ICU) to calculate summary HRs using a random-effects meta-analysis.

**Results:**

Within MIMIC-IV, 1015 patients (24%) died during an average follow-up period of 15.5 days. The fully adjusted HRs and 95% CIs for T2 and T3, relative to T1, were 1.31 (95% CI 1.08–1.58) and 1.54 (95% CI 1.24–1.90), respectively. When grouped by corrected anion gap, the results remained statistically significant. In the meta-analysis, the summary HRs and 95% CIs for T2 and T3 were 1.24 (95% CI 1.08–1.43) and 1.55 (95% CI 1.33–1.82), respectively.

**Conclusions:**

Both the anion gap and corrected anion gap were associated with in-hospital mortality regardless of specific diseases or medical specialties.

## Introduction

Patients admitted to the intensive care unit (ICU) are at a high risk of death, making it crucial to assess the degree of severity to enhance ICU quality [1]. Determining the mortality risk in critically ill patients, who have diverse diagnoses and comorbidities, is challenging. Various prognostic biomarkers exist to estimate mortality among ICU patients, and these markers assist clinicians in stratifying patients based on the risk of specific outcomes [1].

Acid-base imbalances can indicate disease severity and are associated with poor outcomes, including profound effects on the cardiovascular system. As such, an elevated anion gap can be an initial prognostic indicator in critically ill patients [2–4]. While acid-base disorders intuitively reflect the concentration of unmeasured anions, the calculated anion gap can be unreliable due to the influence of albumin levels, as albumin constitutes a substantial portion of the unmeasured anions [5]. Given that hypoalbuminemia is commonly observed in ICU patients, some researchers have recommended using the corrected anion gap for albumin in all critically ill patients [6, 7].

Some studies have explored the relationship between the anion gap and mortality in ICU units caring for specific categories of patients with specific types of diseases [3, 7–16]. However, few studies have investigated the relationship between the anion gap, including the corrected anion gap, and mortality in ICU patients not restricted to particular diseases or hospital departments. This study aimed to investigate the predictive value of the anion gap regarding ICU mortality across different anion gap values, using multiple open-access databases, including the Medical Information Mart for Intensive Care IV (MIMIC-IV).

## Materials and methods

### Data source

We extracted data from MIMIC-IV, an open-access, single-center database of critical care units. The MIMIC-IV database comprises ICU patients admitted to Beth Israel Deaconess Medical Center between 2008 and 2019 [17]. We obtained authorization to access the MIMIC-IV database (date of agreement: 2022.8.3). This study was approved by the Institutional Review Board of Dong-A University Hospital (DAUHIRB-EXP-22-032). All patient information in the MIMIC-IV database was anonymized, and the requirement for informed consent was waived.

### Data extraction

We excluded patients with missing data and those under 20 years of age. For patients with multiple ICU admissions, only data from the first ICU admission were considered. Patients with an ICU stay of fewer than 24 hours were also excluded. Data regarding the following variables were collected within the first 24 hours of ICU admission: demographics (age and sex), clinical factors (body mass index [BMI], mean arterial pressure [MAP], pulse rate [PR], and respiratory rate [RR]), laboratory findings (white blood cell [WBC] count, platelet count, hemoglobin, prothrombin time [PT], activated partial thromboplastin time [aPTT], blood urea nitrogen [BUN], glomerular filtration rate [GFR], and anion gap, as well as serum levels of glucose, albumin, total bilirubin, and lactate), and arterial blood gas measurements (pH, partial pressure of oxygen [pO_2_], and partial pressure of carbon dioxide [pCO_2_]). Means were calculated for any laboratory values measured multiple times within the initial 24 hours. The Charlson Comorbidity Index (CCI) scores were determined based on underlying diseases using ICD-9 codes, and APACHE (Acute Physiology and Chronic Health Evaluation) III scores were also calculated. Data were collected using SQL (Structured Query Language) via Google BigQuery on the Google Cloud Platform (Alphabet Inc., Mountain View, CA, USA). The study’s final participant count was 4229.

### Definition of the anion gap and outcome

The anion gap was determined using the following formula: [Na^+^] + [K^−^]–[Cl^−^]–[HCO_3_^−^] [18]. Study patients were divided into tertile groups (T1–T3) based on their anion gaps. Additionally, the corrected anion gap was computed as follows: corrected anion gap = anion gap + 2.5 x [4.4-albumin (g/dL)] [19]. The study’s primary endpoint was in-hospital mortality. Participants were tracked from the day of ICU admission to either the day of in-hospital death or discharge, whichever occurred first.

### Statistical analysis

Baseline characteristics of the study sample were evaluated as means with standard deviations for continuous variables and as numbers with percentages for categorical variables. Comparisons among study groups were performed using analysis of variance for continuous variables and the chi-squared test or Fisher’s exact test for categorical variables. The in-hospital mortality outcome was calculated by dividing the number of deaths by the total follow-up duration (person-days). Survival probability based on the anion gap group was estimated using the Kaplan-Meier method and evaluated using the log-rank test. To evaluate the association between the anion gap and in-hospital mortality, hazard ratios (HRs) and 95% confidence interval (CIs) for in-hospital mortality were calculated using Cox proportional hazards modeling. Both crude and multivariable-adjusted models were analyzed: Model 1 adjusted for age, sex, BMI, CCI, and APACHE III score; Model 2 adjusted for age, sex, BMI, CCI, APACHE III score, MAP, PR, and RR; and Model 3 (fully adjusted) adjusted for age, sex, BMI, CCI, APACHE III score, MAP, PR, RR, WBC count, platelet count, hemoglobin, PT, aPTT, BUN, GFR, glucose, albumin, total bilirubin, lactate, arterial blood pH, pO_2_, and pCO_2_ level. Subgroup analyses were performed to further evaluate the reliability of the results in terms of in-hospital mortality. We analyzed subgroups according to sex (male and female) and age (≥60 years and <60 years). Moreover, our findings were validated using two additional open databases of critical care units: (1) the MIMIC-III database from the Beth Israel Deaconess Medical Center, spanning 2001 through 2012 [20]; and (2) the eICU (Electronic ICU) Collaborative Research Database, collected from 206 US hospitals between 2014 and 2015 [21]. We extracted the subjects from these two databases using the same criteria and variables described above. The HRs and 95% CIs for in-hospital mortality in each database were calculated using multivariable-adjusted Cox proportional hazards modeling. The three database-level HRs were then combined to calculate summary HRs through a random-effects meta-analysis. All statistical analyses were performed using SPSS Statistics for Windows, version 22 (IBM Corp., Armonk, NY, USA) and R, version 4.3.0 (R Foundation for Statistical Computing, Vienna, Austria). A *p*-value < 0.05 was considered statistically significant.

## Results

### Baseline characteristics of the study sample

The baseline characteristics of the study patients, divided into three groups based on anion gap values, are shown in Table 1. The mean anion gap values for the three groups were 11.8, 15.4, and 21.4, respectively. A higher anion gap was associated with higher BMI, PR, and RR. In addition, a higher anion gap was associated with higher WBC count, PT, aPTT, and BUN, as well as serum levels of glucose, total bilirubin, potassium, and lactate. Conversely, a higher anion gap was associated with lower GFR, sodium, chloride, bicarbonate, pO_2_, and pCO_2_ values. A higher proportion of elevated CCI and APACHE III scores were observed in the third tertile group.

**Table 1:** Baseline characteristics of the study sample.

Anion gap group (Total = 4229)	First tertile (T1, n = 1410)	Second tertile (T2, n = 1410)	Third tertile (T3, n = 1409)	*p*-value
Age (years)	62.5 ± 16.6	62.7 ± 16.5	62.3 ± 16.6	0.81
Sex (male, %)	845 (59.9%)	842 (59.7%)	860 (61.0%)	0.74
Body mass index (kg/m^2^)	29.2 ± 7.8	29.6 ± 8.1	30.1 ± 8.5	0.003
Mean arterial pressure (mmHg)	77.7 ± 9.3	78.4 ± 10.1	76.2 ± 10.6	<0.001
Pulse rate (beats/min)	85.7 ± 16.5	88.8 ± 17.7	92.7 ± 17.9	<0.001
Respiratory rate (cycles/min)	19.2 ± 3.9	20.3 ± 4.0	21.8 ± 4.5	<0.001
White blood cell count (10^9^/L)	12.8 ± 7.0	13.9 ± 7.7	16.0 ± 13.5	<0.001
Platelet count (×10^9^/L)	195.0 ± 104.1	201.1 ± 110.3	190.3 ± 109.7	0.25
Hemoglobin (g/dL)	10.8 ± 1.9	11.1 ± 2.2	10.7 ± 2.3	0.21
Prothrombin time (s)	15.9 ± 6.5	16.5 ± 8.4	20.3 ± 12.4	<0.001
Activated partial thromboplastin time (s)	39.9 ± 19.6	41.6 ± 21.6	46.1 ± 23.1	<0.001
Blood urea nitrogen (mmol/L)	20.9 ± 13.3	27.5 ± 18.5	43.2 ± 30.3	<0.001
Glomerular filtration rate (mL/min/1.73 m^2^)	86.9 ± 47.3	68.6 ± 44.5	41.7 ± 32.6	<0.001
Glucose (mg/dL)	140.4 ± 51.7	157.8 ± 60.9	180.6 ± 83.5	<0.001
Albumin (g/dL)	3.1 ± 0.7	3.2 ± 0.7	3.1 ± 0.7	0.02
Total bilirubin (mg/dL)	1.3 ± 2.3	1.6 ± 3.5	3.1 ± 6.6	<0.001
Sodium (mmol/L)	139.2 ± 4.6	138.7 ± 5.2	138.2 ± 5.5	<0.001
Potassium (mmol/L)	4.2 ± 0.6	4.3 ± 0.6	4.5 ± 0.8	<0.001
Chloride (mmol/L)	106.3 ± 5.7	104.7 ± 6.1	102.2 ± 6.7	<0.001
Bicarbonate (mmol/L)	24.1 ± 4.2	21.9 ± 3.8	18.2 ± 4.2	<0.001
Lactate (mmol/L)	1.9 ± 1.1	2.4 ± 1.5	4.1 ± 3.2	<0.001
Anion gap (mmol/L)	11.8 ± 1.4	15.4 ± 0.9	21.4 ± 4.3	<0.001
pH	7.4 ± 0.1	7.4 ± 0.1	7.3 ± 0.1	<0.001
pO_2_ (mmHg)	178.0 ± 83.5	163.2 ± 76.6	156.0 ± 73.4	<0.001
pCO_2_ (mmHg)	43.5 ± 10.4	41.1 ± 9.2	37.9 ± 9.3	<0.001
Cardiovascular disease (%)	193 (13.7%)	221 (15.7%)	190 (13.5%)	0.19
Congestive heart failure (%)	365 (25.9%)	428 (30.4%)	492 (34.9%)	<0.001
Myocardial infarction (%)	265 (18.8%)	293 (20.8%)	314 (22.3%)	0.07
Chronic obstructive pulmonary disease (%)	394 (27.9%)	352 (25.0%)	318 (22.6%)	0.004
Renal disease (%)	170 (12.1%)	264 (18.7%)	402 (28.5%)	<0.001
Cancer (%)	162 (11.5%)	174 (12.3%)	194 (13.8%)	0.18
Hypertension (%)	628 (44.5%)	628 (44.5%)	565 (40.1%)	0.02
Diabetes (%)	308 (21.8%)	383 (27.2%)	504 (35.8%)	<0.001
Rheumatoid arthritis (%)	45 (3.2%)	56 (4.0%)	44 (3.1%)	0.39
Charlson Comorbidity Index	5.1 ± 2.8	5.5 ± 2.9	6.0 ± 3.0	<0.001
APACHE III score	56.8 ± 25.4	65.4 ± 28.4	83.6 ± 31.1	<0.001
Length of stay for ICU (days)	7.0 ± 7.2	7.9 ± 8.0	8.3 ± 8.4	<0.001
Length of stay for hospital (days)	14.4 ± 12.8	16.2 ± 22.5	15.7 ± 15.5	0.04
Death (%)	185 (13.1%)	306 (21.7%)	524 (37.2%)	<0.001

Data are expressed as mean ± standard deviation or number (%).

### Association between anion gap and in-hospital mortality

**Fig 1:**
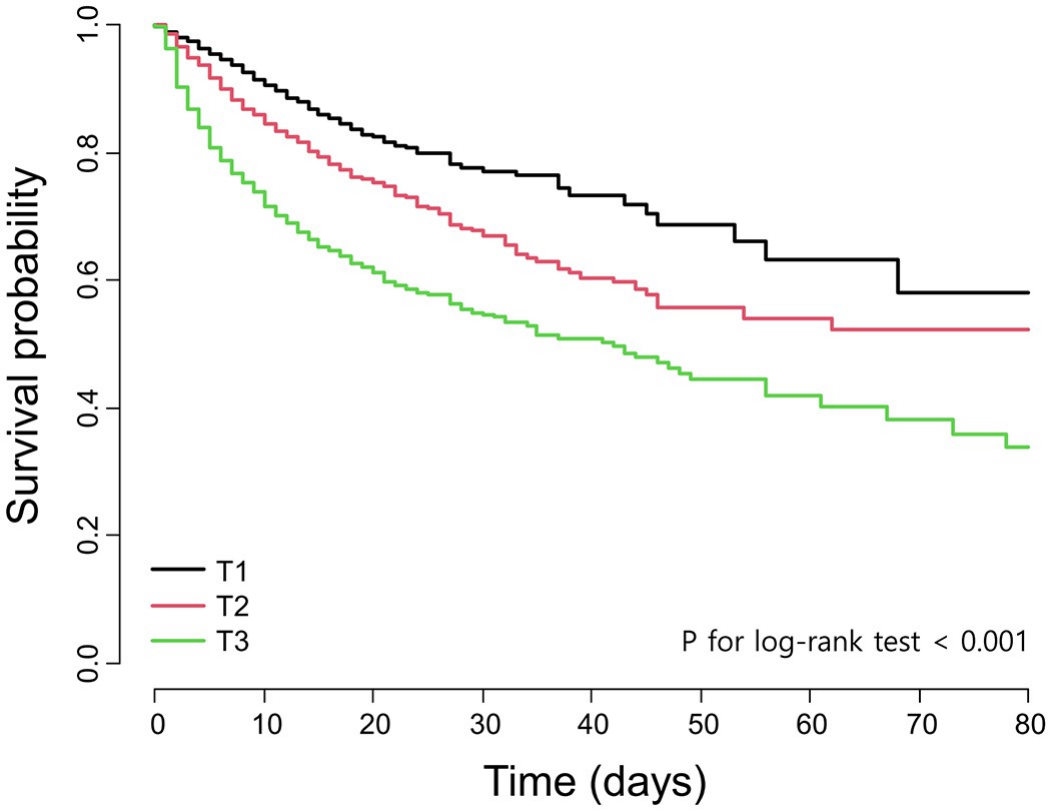
Kaplan-Meier estimates of survival probability according to anion gap tertile. T, tertile.

Of the study sample, 1015 patients died during a mean follow-up of 15.46 days. Fig 1 illustrates the Kaplan-Meier curves; log-rank tests indicated that in-hospital mortality was higher in association with higher anion gap values. As per Table 2, a significant association between the anion gap and in-hospital mortality was observed in the Cox proportional hazards analysis. The fully adjusted HRs and 95% CIs for T2 and T3, relative to T1, were 1.31 (95% CI 1.08–1.58) and 1.54 (95% CI 1.24–1.90), respectively. When the corrected anion gap values were analyzed, the fully adjusted HRs and 95% CIs remained significant: 1.23 (95% CI 1.01–1.49) and 1.47 (95% CI 1.18–1.84), respectively, for T2 and T3 relative to T1.

**Table 2:** Hazard ratios and 95% confidence intervals for in-hospital mortality according to anion gap tertile.

	Events	Follow-up duration (person-days)	Incidence rate (per 1000 person-years)	Hazard ratio (95% confidence interval)							
				Crude	*p*-value	Model 1	*p*-value	Model 2	*p*-value	Model 3	*p*-value
**Anion gap**											
T1	185	20,296	9.12	1.00		1.00		1.00		1.00	
T2	306	22,912	13.36	1.57 (1.31,1.89)	<0.001	1.37 (1.14, 1.64)	0.001	1.35 (1.12, 1.62)	0.002	1.31 (1.08, 1.58)	0.006
T3	524	22,152	23.65	2.74 (2.31, 3.24)	<0.001	1.90 (1.59, 2.26	<0.001	1.84 (1.54, 2.21)	<0.001	1.54 (1.24, 1.90)	<0.001
**Corrected anion gap**											
T1	184	19,037	9.67	1.00		1.00		1.00		1.00	
T2	290	22,898	12.66	1.41 (1.17–1.69)	<0.001	1.17 (0.97, 1.42)	0.09	1.17 (0.97, 1.41)	0.10	1.23 (1.01, 1.49)	0.04
T3	541	23,425	23.09	2.62 (2.21, 3.09	<0.001	1.64 (1.36, 1.97)	<0.001	1.61 (1.34, 1.94)	<0.001	1.47 (1.18, 1.84)	0.001

T, tertile.

Model 1, adjusted for age, sex, BMI, CCI, and APACHE III score.

Model 2, adjusted for age, sex, BMI, CCI, APACHE III score, MAP, PR, and RR.

Model 3, adjusted for age, sex, BMI, CCI, APACHE III score, MAP, PR, RR, WBC count, platelet count, hemoglobin, PT, aPTT, BUN, GFR, glucose, albumin, total bilirubin, lactate, arterial blood pH, pO_2_, and pCO_2_ level.

### Subgroup analysis according to sex and age

**Fig 2:**
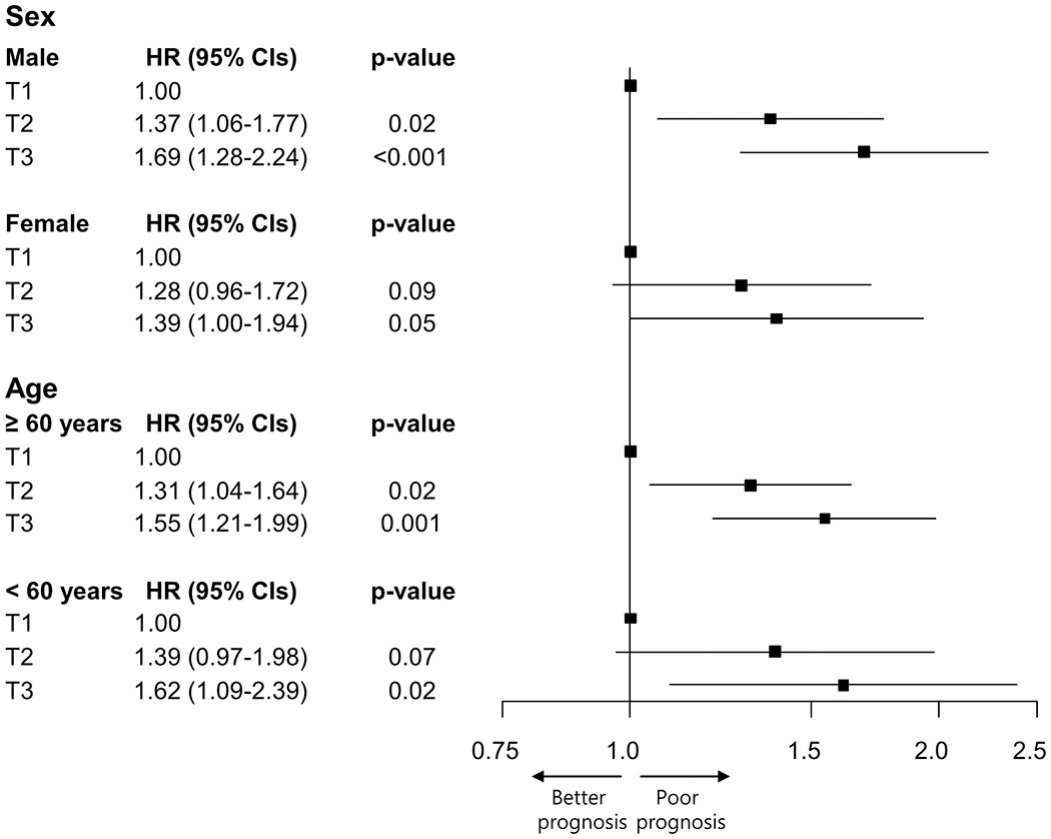
Hazard ratio and 95% confidence intervals for in-hospital mortality in the subgroup analysis. HR, hazard ratio; CIs, confidence intervals.

The analysis according to sex and age, in relation to anion gap value, is depicted in Fig 2. When categorized by sex, the HRs and 95% CIs for in-hospital mortality were significant, particularly for males: 1.37 (95% CI 1.06–1.77) and 1.69 (95% CI 1.28–2.24), respectively, for T2 and T3 relative to T1. In the age-based subgroups, all of the HRs and 95% CIs for in-hospital mortality were statistically significant.

### Meta-analysis using other open databases

**Fig 3:**
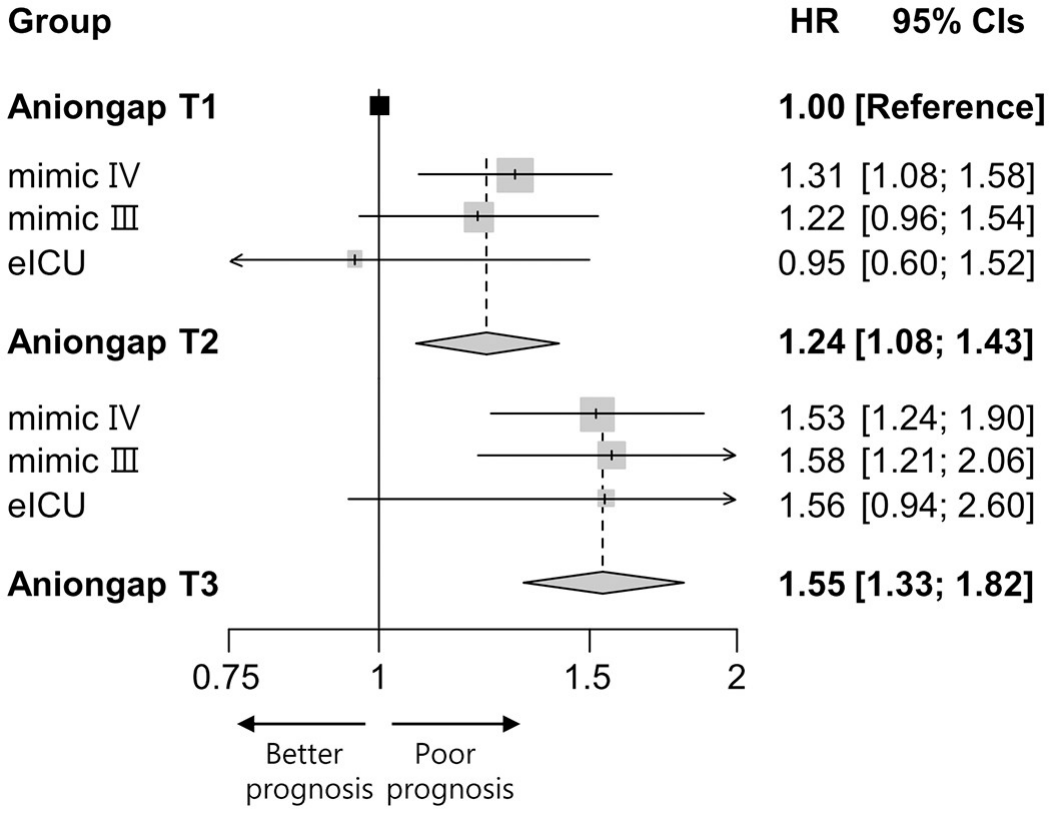
Meta-analysis of in-hospital mortality according to anion gap. HR, hazard ratio; CIs, confidence intervals.

The MIMIC-III and eICU databases contained 3069 and 697 patients, respectively. Within the MIMIC-III database, the mean anion gap values across the three groups were 11.4, 14.8, and 20.3, respectively. In the eICU database, the values were 7.3, 11.7, and 18.8, respectively. Summary HRs and CIs were calculated after pooling the data from all three databases. The meta-analysis revealed a significant association between anion gap values and in-hospital mortality in Fig 3. For the anion gap groups, the summary HRs and 95% CIs for T2 and T3, relative to T1, were 1.24 (95% CI 1.08–1.43) and 1.55 (95% CI 1.33–1.82), respectively.

## Discussion

This study demonstrated that higher anion gap values were associated with higher in-hospital mortality in the MIMIC-IV database. Even after adjusting for multiple covariates, a higher anion gap remained a significant predictor of in-hospital mortality. When the corrected anion gap was used to adjust for albumin levels, the results remained consistent. A meta-analysis incorporating the MIMIC-III and eICU databases also showed the anion gap to be associated with in-hospital mortality.

Many previous studies have used the anion gap to predict mortality rates for specific patient groups or designated hospital departments [3, 7–16]. These studies have demonstrated associations in populations such as patients with cardiogenic shock, cardiac disease, post–cardiac arrest complications, disseminated intravascular coagulation, postoperative status, and advanced kidney disease. Ji and Peng demonstrated a positive correlation between serum anion gap values and all-cause mortality among unselected adult patients using only the MIMIC-III database [22]. While many studies have concentrated on specific patient groups and relied on single databases or a limited set of covariates, our research has delved into the association between the anion gap (including the corrected anion gap) and in-hospital mortality among general ICU patients. This was achieved by using multiple data sources (encompassing as many covariates as possible): the MIMIC-IV, MIMIC-III, and eICU databases.

The anion gap quantifies the difference between cations(Na^+^ and K^+^) and anion (Cl^-^ and HCO3^-^) concentrations in the blood [18]. In a theoretically electro-neutral human body, unquantifiable plasma anions maintain an anion gap ranging from 4 to 12 mmol/L under normal conditions. Deviations from this range suggest possible imbalances between measured and unmeasured ions. Historically, the anion gap has been used as an indicator for evaluating acid–base imbalances and metabolic abnormalities [23]. An elevated serum anion gap typically points to increased production of organic acid anions or diminished anion excretion. While the exact physiological mechanisms underlying acid–base disturbances are not fully understood, acid–base equilibrium is known to reflect disease prognosis and severity, including organ dysfunction—a vital concern for ICU patients [24, 25].

In our analysis, lactate values were also considered as a covariate; however, even after this adjustment, the anion gap’s significance persisted. This might be because of the presence of other unmeasured anions, such as ketones and pyruvate. ICU patients often exhibit elevated metabolic rates, marked by sympathetic activation, accelerated glycolysis, and increased serum concentrations of unmeasured anions, including lactate and ketones [26, 27]. Furthermore, impaired kidney function is common in ICU patients, and this can lead to an accumulation of unmeasured anions due to the build-up of acidic compounds [14]. Collectively, these findings strongly suggest that a higher anion gap may be associated with prognosis.

This study had several limitations. Firstly, it was retrospective in nature, which can introduce selection bias and misclassification bias. Secondly, the identification of underlying diseases relied on ICD codes, which may introduce misclassification bias. Lastly, while we incorporated numerous potential covariates, there may have been additional variables not considered. Consequently, future prospective studies are warranted to validate the relationship between the anion gap and mortality.

## Conclusions

This study demonstrated an association between an increased anion gap levels and elevated in-hospital mortality. Furthermore, the anion gap might hold potential as a prognostic indicator for mortality risk in a broad patient population, which aligns with the overarching goal of identifying measurable biomarkers to forecast patient outcomes and guide treatment approaches.

## Supporting information

S1 Checklist Human participants research checklist. (DOCX)
